# A contribution to Asian *Afidentula* Kapur (Coleoptera, Coccinellidae, Epilachnini)

**DOI:** 10.3897/zookeys.516.9665

**Published:** 2015-08-06

**Authors:** Xingmin Wang, Wioletta Tomaszewska, Shunxiang Ren

**Affiliations:** 1 Engineering Research Centre of Biological Control, Ministry of Education, South China Agricultural University, Guangzhou, 510642 China; 2 Museum and Institute of Zoology, Polish Academy of Sciences, Wilcza 64, 00-679 Warszawa

**Keywords:** Entomology, taxonomy, Cucujoidea, *Afidentula*, *Afidenta*, new species

## Abstract

Two new species of *Afidentula*, *Afidentula dentata* **sp. n.** and *Afidentula jinpingensis* **sp. n.** are described from China. *Afissa siamensis* Dieke is moved to *Afidentula* **comb. n.**. All three species are described and illustrated, and a distribution map is given. A key to Asian species of *Afidentula* is updated. Diagnostic similarities and differences between *Afidentula* and *Afidenta* are discussed and illustrated.

## Introduction

The genera *Afidentula* Kapur, 1958 and *Afidenta* Dieke, 1947 belong to the tribe Epilachnini Mulsant, 1846, the group of phytophagous Coccinellidae. The taxonomy and nomenclatural history of species of both genera have been confused for decades.

The genus *Afidenta* was established by Dieke (1947) for species having bifid claws with a sharp basal tooth and sixth abdominal ventrite of female not longitudinally divided. *Afidenta mimetica* Dieke (=*Afidenta misera* (Weise)) was designated as the type species. Other two species, *Afidenta minima* (Gorham, 1894) and *Afidenta bisquadripunctata* (Gyllenhal in Schönherr, 1808) were placed in this genus at the same time, although Dieke noted that the mandibles and male genitalia of *Afidenta bisquadripunctata* were different from the type species of *Afidenta*.

Kapur (1958) established the genus *Afidentula* with *Epilachna manderstjernae* Mulsant as the type species and distinguished it from *Afidenta* by the antennae subequal to the width of the head with a relatively thick and compact club and subtriangular mandibles with three teeth and without any additional denticulations or serrations. Kapur (1958) also pointed that both *Afidenta minima* and *Afidenta bisquadripunctata* should not belong to *Afidenta* but transferred only *Afidenta minima* to *Afidentula*. Subsequently, *Afidentula himalayana* Kapur, 1963 from India and *Afidentula thanhsonensis* Hoang, 1977 from Vietnam have been described, and several other mainland Asian species were added to that genus, e.g. *Epilachna stephensi* was transferred to *Afidentula* by Booth and Pope (1989). Bielawski (1963) transferred the Papuan *Epilachna aruensis* Crotch to *Afidentula* and Bielawski (1963, 1965) and Jadwiszczak (1986) added further new species from New Guinea.

Li in Li and Cook (1961) described *Afidenta arisana* from Taiwan, which was moved to *Afissula* Kapur by Zeng (1995). Pang and Mao (1979) transferred *Afissa siamensis* Dieke into *Afidenta* and moved *Afidenta bisquadripunctata* into *Afidentula*.

Chazeau (1975, 1976) studied African Epilachninae, and described 29 new species, which included nine species of *Afidenta*. Fürsch (1986) revised species of *Afidenta* describing five new species and included 25 species but not Chazeau’s (1975, 1976) species.

Jadwiszczak and Węgrzynowicz (2003) listed 39 species belonging to *Afidenta* (of which 37 have been distributed in Africa and two in Asia) and 18 species of *Afidentula* (11 species distributed in mainland Asia and seven in New Guinea and Aru Island).

Tomaszewska and Szawaryn (2013), and Szawaryn and Tomaszewska (2013) revised Asian and Papuan species of *Afidentula*. They concluded that the mainland species of the *Afidentula* form uniform group which can be characterized by: comparatively small body, brown colour with black markings on elytra, compact and short mandibles provided with three apical teeth of which only middle one is sometimes weakly serrated, maxilla with basistipes and mediastipes separated entirely or almost so, terminal labial palpomere shorter than subterminal one, tibial spurs absent, tarsal claw with basal tooth present, and sternite VIII in females undivided. Species from New Guinea and Aru Island are considerably different having among others the body much larger and entirely black or black with orange spots on elytra, mandibles large and thin laterally with apical and subapical teeth, often additionally serrated, elytral epipleura complete (incomplete in *Afidentula*), the distance between antennal sockets about three or four times greater than a distance between antennal socket and inner margin of eye (in *Afidentula* this distance is about twice as great), coxites with styli and the tegmen with stout parameres. For New Guinean species Szawaryn and Tomaszewska (2013) proposed a new genus *Papuaepilachna* and for *Afidentula aruensis* form Aru Island a new genus *Lalokia*.

Szawaryn et al. (2015) conducted phylogenetic research on Epilachnini based on molecular and morphological data. According to this study, both *Afidenta* and *Afidentula* have not been recovered as monophyletic groups and each of them has been redefined. Studied species of *Afidenta* from Africa formed monophyletic clade with Asian mainland species of *Afidentula* and exclusion of the Papuan species from *Afidentula* has been confirmed by the study. From among two species of *Afidenta* from Asia, the type species (*Afidenta misera*) was studied and it formed a separate clade by itself, based on the following combination of characters: ventral surface of the mandible densely tuberculate, galea transversely oval, terminal palpomere of labium distinctly narrower than penultimate one, metaventral postcoxal lines joined or almost so on metaventral process, forming somewhat w-shaped line along discrimen, male tergite VIII rounded apically and styli absent. The definition of *Afidentula* has been extended after inclusion of African species of *Afidenta* and some Malagasy *Epilachna* and *Henosepilachna*, and it has been characterized by the following combination of characters: gular sutures shorter than half-length of gula, mandibular incisor edge without teeth, terminal maxillary palpomere weakly elongate, expanded apically, labial apical palpomere distinctly narrower than penultimate palpomere and styli absent.

Based on the results of the phylogenetic analyses of Szawaryn et al. (2015), the present paper describes two new species of *Afidentula* from China, *Afidentula dentata* sp. n. and *Afidentula jinpingensis* sp. n. The study of *Afidenta siamensis* permits the move of this species from *Afidenta* to *Afidentula* as *Afidentula siamensis* comb. n., confirming with this that *Afidenta* now includes only one species.

## Material and methods

The external morphology was observed with a dissecting stereoscope (SteREO Discovery V20, Zeiss and Leica Mz Apo). The following measurements were made with an ocular micrometer: total length, length from apical margin of clypeus to apex of elytra (TL); total width, width across both elytra at widest part (TW=EW); height, from the highest part of the beetle to elytral outer margins (TH); head width in front view, widest part (HW); pronotal length, from the middle of anterior margin to margin of basal foramen (PL); pronotal width at widest part (PW); elytral length, along suture, from the apex to the base including scutellum (EL). Male and female genitalia were dissected, cleared in 10% solution of NaOH by boiling for several minutes, and examined with an Olympus BX51 and Leica compound microscope.

Morphological characters were photographed with digital cameras (AxioCam HRc and Coolsnap–Pro*cf* & CRI Micro*Color), connected to the dissecting microscope. The software AxioVision Rel. 4.8 and Image-Pro Plus 5.1 were used to capture images from both cameras, and photos were cleaned up and laid out in plates with Adobe Photoshop CS 8.0.

Coccinellidae morphological terms follow Ślipiński (2007) and Ślipiński and Tomaszewska (2010). Type specimens designated in the present paper are deposited at SCAU-the Department of Entomology, South China Agriculture University, Guangzhou, China.

## Taxonomy

### Genus Afidentula

Taxon classificationAnimaliaColeopteraCoccinellidae

Kapur

Afidentula Kapur, 1958: 324. Type species: *Epilachna manderstjernae* Mulsant, 1853 (by original designation). – Jadwiszczak and Węgrzynowicz 2003; Kovár 2007; Ren et al. 2009; Tomaszewska and Szawaryn 2013; Szawaryn et al. 2015. part of *Afidenta* Dieke, 1947; Szawaryn et al. 2015. part of *Epilachna* Chevrolat in Dejean, 1837; Szawaryn et al. 2015. part of *Henosepilachna* Li in Li & Cook, 1961; Szawaryn et al. 2015.

#### Diagnosis and comments.

**Figure 1.**
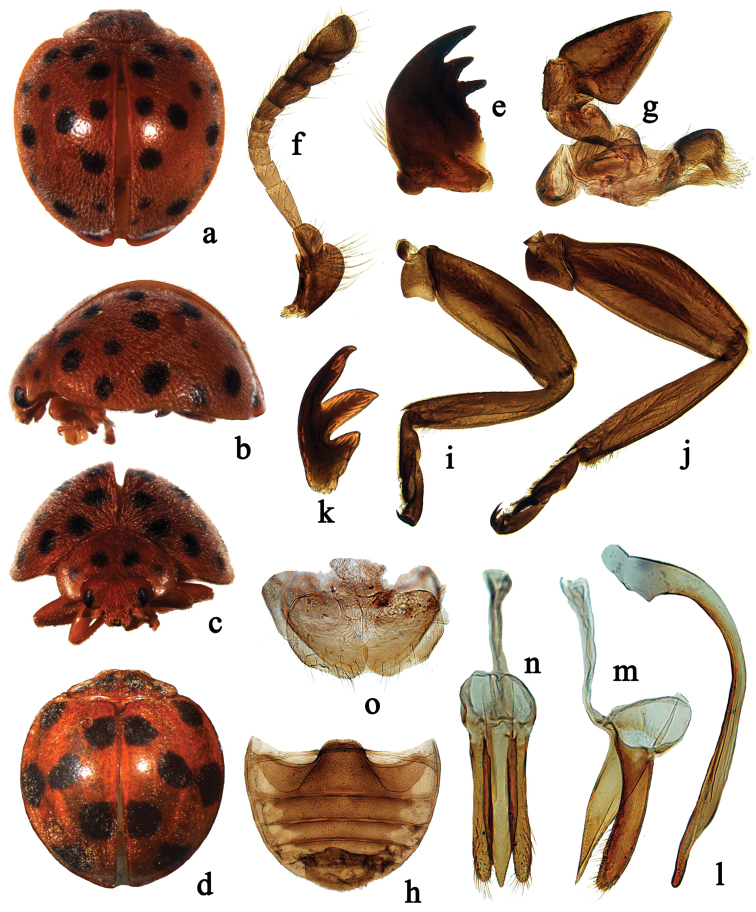
*Afidenta misera* (Weise, 1909). (**a, d**) dorsal habitus **b** lateral habitus **c** frontal habitus **e** mandible **f** antenna **g** maxilla **h** abdomen **i** front leg **j** hind leg **k** tarsal claw **l–n** male genitalia: **l** penis **m** tegmen, lateral view **n** tegmen, ventral view **o** ovipositor.

**Figure 2.**
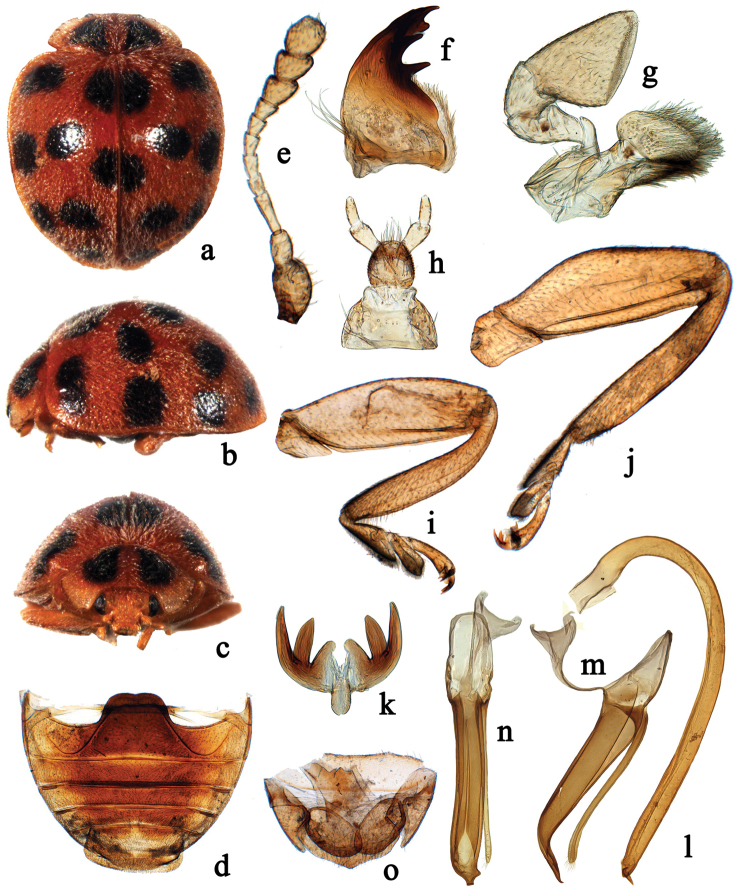
*Afidentula siamensis* (Dieke, 1947), comb. n. **a** dorsal habitus **b** lateral habitus **c** frontal habitus **d** abdomen **e** antenna **f** mandible **g** maxilla **h** labium **i** front leg **j** hind leg **k** tarsal claw **l–n** male genitalia: **l** penis **m** tegmen, lateral view **n** tegmen, ventral view **o** ovipositor.

**Figure 3.**
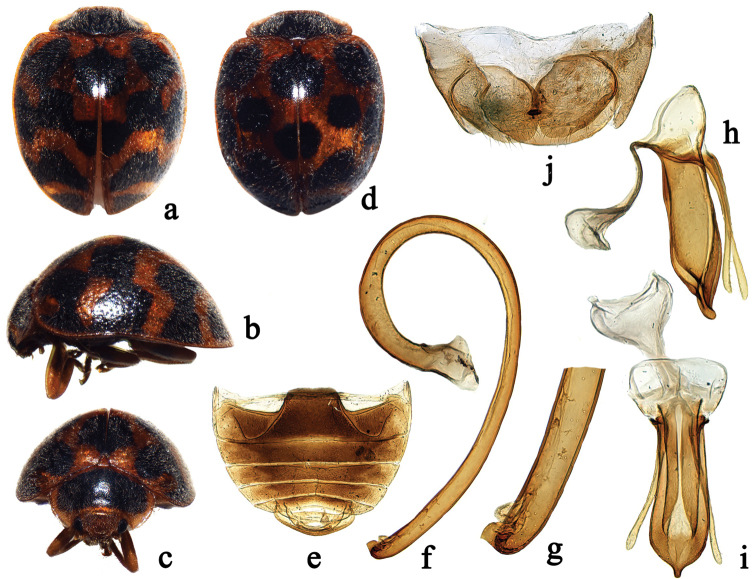
*Afidentula dentata* sp. n. (**a, d**) dorsal habitus **b** lateral habitus **c** frontal habitus **e** abdomen **f–h** male genitalia: **f** penis **g** apex of penis **h** tegmen, lateral view **i** tegmen, ventral view **j** ovipositor.

**Figure 4.**
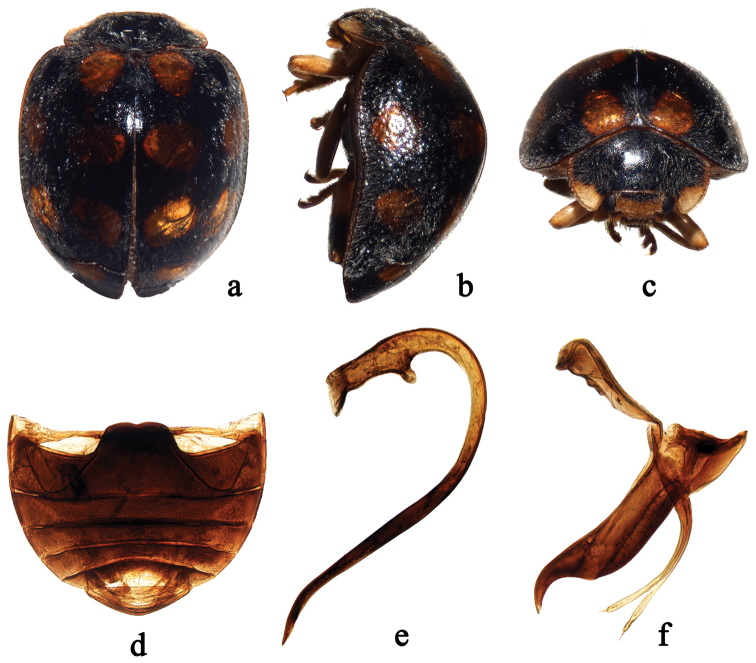
*Afidentula jinpingensis* sp. n. **a** dorsal habitus **b** lateral habitus **c** frontal habitus **d** abdomen **e–f** male genitalia: **e** penis **f** tegmen, lateral view.

Species of *Afidentula* are most similar to *Afidenta* by the general body shape and colouration (Figs 1a–d, 2a–c, 3a–d, 4a–c), bifid tarsal claws with a large basal tooth (Figs 1k, 2k), abdominal sternite VIII in female not divided longitudinally and female genitalia with oval coxites lacking styli (Figs 1h, o, 2d, o, 3e, j, 4d). *Afidentula* in the present sense (Szawaryn et al. 2015) constitutes morphological pretty diverse group, especially after inclusion of some species of former *Epilachna* and *Henosepilachna* from Madagascar. The Asian species of *Afidentula*, however, can be easily distinguished from *Afidenta* (and other Epilachnini genera) by the following combination of characters: mandibular incisor edge smooth (incisor edge microdenticulate in *Afidenta*, Fig. 1e), ventral surface of incisor edge without tubercles, terminal labial palpomere narrower and shorter than penultimate one (narrower but as long as penultimate one in *Afidenta*), metaventral postcoxal lines joined on metaventral process in form of straight line (forming somewhat w-shaped line along discrimen in *Afidenta*), tibial spurs absent (present in *Afidenta*, Figs 1i-j), abdominal postcoxal lines complete or almost so (distinctly incomplete in *Afidenta*, Fig. 1h), tegminal strut triangularly expanded apically (simple in *Afidenta*, Fig. 1m–n), parameres shorter than penis guide and very narrow (in *Afidenta*, parameres as long as penis guide and much broader than in *Afidentula*, Fig. 1m–n).

*Afidentula* is also similar to *Afissa* Dieke (=*Afissula* Kapur) in general appearance, but it can be separated by having antennae distinctly shorter than width of the head and with at least antennomeres 7 and 8 subquadrate (in *Afissa* antennae are longer than width of head and have antennomeres 3–8 elongate) and tibiae without apical spurs (tibial spurs present in *Afissa*).

Monographic revision of all Epilachnini genera based on the results of phylogenetic analysis is in preparation (Tomaszewska and Szawaryn, in prep.) and richly illustrated; detailed descriptions of all genera will be provided there.

#### Distribution.

Asia and Africa.

#### Key to the Asian species of *Afidentula*

(based on Tomaszewska and Szawaryn 2013)

**Table:** 

1	Background of elytra black, covered with yellowish orange, round spots (Fig. 4a–c)	***Afidentula jinpingensis* sp. n.**
–	Background of elytra brown, covered with black spots or bands	**2**
2	Lateral and basal margins of elytra black; disk of each elytron with three round-oval, black spots	***Afidentula minima* (Gorham)**
–	Lateral and basal margins of elytra brown like elytral background; each elytron with more than three spots, rarely some of them may be fused and arranged in transverse bands	**3**
3	Each elytron with four black spots	**4**
–	Each elytron with more than four spots or with transverse bands	**6**
4	Body 1.13 times as long as wide, heart-shaped; elytra chestnut brown; epipleura about 3.5 times wider than metepisternum	***Afidentula semisqualens* Tomaszewska & Szawaryn**
–	Body 1.24–1.43 times as long as wide, oval; elytra reddish brown or orange; epipleura at most 2.65 times wider than metepisternum	**5**
5	Labial palpomere 2 at most 1.15–1.20 times longer than terminal palpomere; prosternal process about 0.28–0.30 times as wide as longest procoxal diameter, truncate apically; penis guide with sharp tooth in apical fourth and short incision at apex	***Afidentula bisquadripunctata* (Gyllenhal)**
–	Labial palpomere 2 1.35–1.50 times longer than terminal palpomere; prosternal process about 0.34–0.36 times as wide as longest procoxal diameter, weakly rounded apically; penis guide with blunt tooth in apical fourth and without incision at apex	***Afidentula thanhsonensis* Hoàng**
6	Body larger, 4.90–5.33 mm long; long-oval, 1.38–1.43 times as long as wide; elytra 1.17–1.20, almost parallel-sided	**7**
–	Body smaller, 2.83–4.80 mm long; short-oval, 1.16–1.33 times as long as wide; elytra 0.97–1.13 times as long as wide, oval	**8**
7	Head black; mesoventral process about 0.55 times as wide as mesocoxal diameter; meso- and metaventrite without distinct grooves behind anterior raised borders	***Afidentula quindecemguttata* (Dieke)**
–	Head red or reddish brown; mesoventral process about 0.65 times as wide as mesocoxal diameter; with distinct groove behind anterior raised border of mesoventrite and behind raised border of metaventral process	***Afidentula himalayana* Kapur**
8	Elytron with medio-anterior spot distant from scutellum and suture; body 2.83–3.68 mm long	***Afidentula manderstjernae* (Mulsant)**
–	Elytron with medio-anterior spot touching at least suture; body 3.85–4.80 mm long	**9**
9	Medio-anterior spot on each elytron touching one another along suture but constitute distinct separate spots; antennomere 4 slightly longer than 5; penis guide in anterior view gradually narrowing from mid length to apex	***Afidentula stephensi* (Mulsant)**
–	Medio-anterior spot on each elytron fused together forming one macula; antennomere 4 not longer than 5 (Figs 2a–b, 3a–d); penis guide in ventral view subparallel or even widening before apex (Figs 2n, 3i)	**10**
10	Antennomere 4 and 5 subequal in length; pronotum with large black spot which almost cover whole surface of pronotum (Fig. 3c); apex of penis with two tooth-shaped processes directed inwardly (Fig. 3f–g)	***Afidentula dentata* sp. n.**
–	Antennomere 4 shorter than 5; pronotum with two, separate large black spots (Fig. 2c); apex of penis with small, sharp process directed outwardly (Fig. 2l)	***Afidentula siamensis* (Dieke)**

### Afidentula siamensis

Taxon classificationAnimaliaColeopteraCoccinellidae

(Dieke, 1947) comb. n.

Figures 2 , 5

Afissa siamensis Dieke, 1947: 127.
Afidenta siamensis : Pang and Mao 1979: 119; Cao 1992: 221; Ren et al. 2009: 250.

#### Diagnosis.

This species is most similar to *Afidentula dentata* and *Afidentula stephensi* (known from India and Pakistan) but can be distinguished from both by having pronotum with two large black oval spots, apex of penis with small sharp process directed outwardly (Fig. 2a–c, 2l) and apex of penis guide curved outwardly (Fig. 2m–n).

#### Description.

TL: 4.0–4.3 mm, TW: 3.0–3.7 mm, TH: 1.8–2.1 mm, TL/TW: 1.16–1.33; PL/PW: 0.35–0.36; EL/EW: 0.97–1.13; HW/TW: 0.30; PW/TW: 0.77.

Body short oval, dorsum strongly convex, densely pubescent (Fig. 2a–c). Head yellowish brown. Pronotum yellowish brown except anterior corners yellowish white, with two large black, triangularly-oval spots. Scutellum yellowish brown. Elytra yellowish brown, with 14 rounded black spots, arranged as in Fig. 2a–c. Underside yellowish brown, except metaventrite and middle area of abdomen black. Epipleura and legs yellow.

Head with frontal punctures moderately large and densely distributed, 0.8–1.0 diameters apart, associated with scattered long setae; interocular distance 0.64 times head width (Fig. 2c). Pronotal disk with fine and densely distributed punctures, distinctly smaller than those on head, 2.0–4.0 diameters apart. Elytra dually punctate; large punctures 1.0–6.0 diameters apart and small ones 1.0–4.0 diameters apart. Surfaces of prosternum and mesoventrite shagreened, with scattered short setae. Metaventrite broad with fine and densely distributed punctures, 2.0–4.0 diameters apart.

Male genitalia. Penis short and stout, strongly curved at base, apex with small and sharp process directed inwardly, capsule inconspicuous (Figs 2l). Tegmen stout (Fig. 2m–n); penis guide in lateral view widest at base and narrowing to apex, strongly curved outwardly at apical 1/4, apex pointed (Fig. 2m); parameres slender, distinctly shorter than penis guide (Fig. 2m); penis guide in ventral view flattened and asymmetrical at apex, lateral margins almost parallel, apex blunt (Fig. 2n).

Female terminalia and genitalia. Proctiger (TX) triangularly pointed at apex. Coxites oval, without styli, apical margin with several setae. (Fig. 2o). Spermatheca not studied.

#### Specimens examined.

**Holotype.** Nan, Siam, Jan. 27/28, Cockerell/ Type No. 57138 USNM/ *Afissa siamensis* Dieke, holotype.

CHINA, Yunnan Prov.: 1 male, Jiluoshan, Xishuangbanna National Natural Reserve, Mengla County, 6.v.2009, Wang XM et al. leg; 1 female, Lafu, Menglian County, 1130m, 7.v.2008, Wang XM et al. leg; Guizhou Prov.: 3 males, Dadugang, Badu Town, Ceheng County, 15.x.2006, Wang XM leg.

#### Distribution.

China: Guizhou, Yunnan; Thailand.

**Figure 5.**
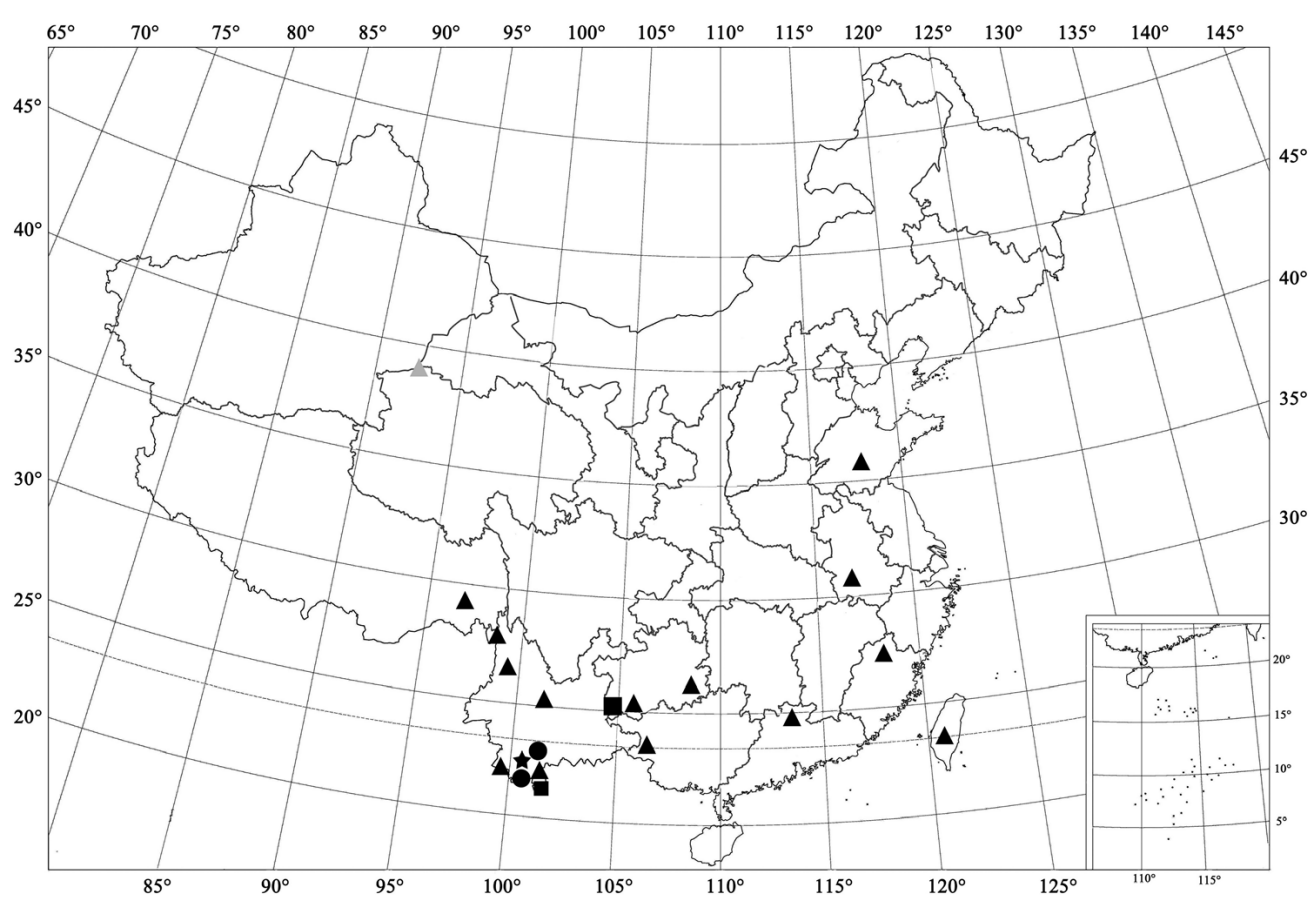
Distribution map. *Afidenta misera* (Weise, 1909) (▲); *Afidentula siamensis* (Dieke, 1947), comb. n. (■); *Afidentula dentata* sp. n. (●); *Afidentula jinpingensis* sp. n. (★).

#### Remark.

Pang and Mao (1979) transferred *Afissa siamensis* Dieke into *Afidenta* without any explanation. However, a detailed examination of *Afidenta siamensis* and *Afidenta misera* left no doubt that they do not belong to a same genus, and that diagnostic characters of *Afidenta siamensis* match *Afidentula*. Thus this species in formally transfered to the genus *Afidentula*.

### Afidentula dentata sp. n.

Taxon classificationAnimaliaColeopteraCoccinellidae

http://zoobank.org/A8E6482A-BBA3-432C-BC20-F072867D7C5F

Figures 3 , 5

#### Diagnosis.

This species is most similar to *Afidentula siamensis* in general appearance and colouration, e.g. having two mutual maculae on elytra along suture (anteriorly and medially) but can be distinguished from the latter by having pronotum with a large black spot which almost covers entire surface of the pronotum leaving only lateral and anterior margins brown (Fig. 3a–d), and apex of penis with two tooth-shaped appendices inwardly (Fig. 3f–g). In *Afidentula siamensis*, pronotum has two large black spots, and apex of penis has a small and sharp process directed outwardly (Fig. 2a–c, l).

#### Description.

TL: 4.20–4.80 mm, TW: 3.40–3.90 mm, TH: 1.90–2.40 mm, TL/TW: 1.23–1.24; PL/PW: 0.42–0.43; EL/EW: 0.97–1.03; HW/TW: 0.31; PW/TW: 0.62.

Body short oval, dorsum strongly convex, densely pubescent (Figs 3a–d). Head yellowish brown. Pronotum mostly black with only lateral and anterior margins yellowish brown (Fig. 3c). Scutellum yellowish brown. Elytra yellowish brown, with 14 rounded black spots arranged as in Figures 3d; spots may connect to each other forming transverse bands (Fig. 3a, b). Underside yellowish brown, except meso-, metaventrite and middle area of abdomen dark brown. Epipleura yellowish brown, except areas close to meso- and metaventrite dark brown. Legs yellow.

Head with frontal punctures fine and densely distributed, 1.0–1.5 diameters apart, associated with scattered long setae; interocular distance 0.67 times head width (Fig. 3c). Pronotal disk with fine and densely distributed punctures, slightly smaller than those on head, 1.0–2.0 diameters apart. Elytral disk dually punctate, large punctures 1.0–6.0 diameters apart and small ones 2.0–4.0 diameters apart. Surfaces of prosternum and mesoventrite shagreened, with scattered short setae. Metaventrite broad with fine and densely distributed punctures, 1.0–2.0 diameters apart.

Male genitalia. Penis stout, strongly curved, apex with two tooth-shaped appendixes directed inwardly, capsule inconspicuous (Fig. 3f–g). Tegmen stout (Fig. 3h–i); penis guide in lateral view short and stout, widest at base, lateral margins almost parallel along basal 4/5, and then suddenly narrowed to apex, apex slightly curved outwardly (Fig. 3h). Parameres slender and almost straight, distinctly shorter than penis guide (Fig. 3h). Penis guide in ventral view flattened and symmetrical, widest at apical 1/10, gradually weakly narrowing to base but strongly narrowing to apex, apex finger-shaped protruded (Fig. 3i).

Female terminalia and genitalia. Proctiger (TX) rounded apically. Coxites oval, without styli, apical margin with small protuberance and several setae (Fig. 3j). Spermatheca not studied.

#### Types.

**Holotype**: male, CHINA, Yunnan Prov.: Menglun, Xishuangbanna National Natural Reserve, Mengla County, 21.viii.2005, Wang XM leg; **Paratypes (110)**: CHINA, Yunnan Prov.: 3 males, same data as holotype; 1 male, Longmen Village, Shangyong Town, Mengla County, 1.v.2008, Wang XM leg; 2 males, Menglun, Xishuangbanna National Natural Reserve, Mengla County, 29.iv.2008, Wang XM leg; 1 female, Yaoqu Villge, Mengla County, 700m, 7–8.v.2009, Ren SX leg; 30 females and males, Jiluoshan, Xishuangbanna National Natural Reserve, Mengla County, 28.iv.2008, Wang XM et al. leg; 11 females and males, Menga Town, Mengla County, 1170m, 12.v.2009, Ren SX et al. leg; 5 females and males, Jiluoshan, Xishuangbanna National Natural Reserve, Mengla County, 6.v.2009, Wang XM et al. leg; 6 females and males, Caiyanghe Natural Reserve, Puer County, 4.v.2009, Wang XM et al. leg.; 7 females and males, Longtan, Ximeng County, 900m, 9-10.v.2008, Wang XM et al. Leg.; 11 females and males, Banhong, Nangunhe National Natural Reserve, 1790m, 14-15.v.2008, Wang XM et al. leg.; 33 females and males, Banlao, Nangunhe National Natural Reserve, 1100m, 16.v.2008, Wang XM et al. leg.

#### Distribution.

China (Yunnan).

#### Etymology.

The specific epithet is formed from the Latin adjective *dentatus*, referring to the apex of penis with two tooth-shaped processes.

### Afidentula jinpingensis sp. n.

Taxon classificationAnimaliaColeopteraCoccinellidae

http://zoobank.org/86095FE9-6635-4AB4-8903-1E926E8A0D88

Figures 4 , 5

#### Diagnosis.

This species can be easily distinguished from all other Asian species of *Afidentula* by having elytra black covered with 12 yellow spots (Fig. 4a).

#### Description.

TL: 4.6–4.8 mm, TW: 3.7–4.0 mm, TH: 2.0–2.2 mm, TL/TW: 1.20–1.24; PL/PW: 0.43–0.46; EL/EW: 1.05–1.08; HW/TW: 0.28; PW/TW: 0.62.

Body short oval, dorsum strongly convex, densely pubescent (Figs 4a–c). Head with frons yellowish brown and occiput black. Pronotum black with anterior corners pale yellow. Scutellum black. Elytra black, each elytron with six yellow spots, arranged as 1-2-2-1. Underside yellowish brown, except metaventrite black. Epipleura yellowish brown at basal 1/3 and dark brown at apical 2/3. Legs yellowish brown.

Head with frontal punctures fine, 1.0–2.0 diameters apart, associated with scattered long setae; interocular distance 0.64 times head width (Fig. 4c). Pronotal disk with fine and densely distributed punctures, slightly larger than those on head, 0.5–1.0 diameters apart. Elytral disk dually punctate; large punctures 1.0–6.0 diameters apart and small ones 1.0–4.0 diameters apart. Surfaces of prosternum and mesoventrite shagreened, with scattered setae. Metaventrite broad with fine and inconspicuous punctures.

Male genitalia. Penis stout, strongly curved, apex simple and pointed, capsule with an expanded outer arm and a small inner one (Fig. 4e). Tegmen stout (Fig. 4f); penis guide in lateral view subparallel along 4/5 of its length and hook-like at apex; apex curved outwardly; parameres extremely slender, distinctly shorter than penis guide.

Female genitalia not known.

#### Types.

**Holotype**: male, CHINA, Yunnan Prov.: Fenshuiling National Natural Reserve, Jingpin County, 1800–2200m, 18.v.2009, Ren SX leg.

#### Distribution.

China (Yunnan).

#### Etymology.

The specific epithet is named after Jingpin County, China, the type locality of this ladybird.

## Supplementary Material

XML Treatment for Afidentula

XML Treatment for Afidentula siamensis

XML Treatment for Afidentula dentata

XML Treatment for Afidentula jinpingensis
